# Transcriptomic Establishment of Pig Macrophage Polarization Signatures

**DOI:** 10.3390/cimb45030151

**Published:** 2023-03-12

**Authors:** Jing Li, Teng Yuan, Anjing Zhang, Peidong Yang, Li He, Keren Long, Chuang Tang, Li Chen, Mingzhou Li, Lu Lu

**Affiliations:** 1 College of Animal Science and Technology, Sichuan Agricultural University, Chengdu 611130, China; 2 Chongqing Academy of Animal Sciences, Chongqing 402460, China

**Keywords:** porcine, macrophage, differentiation, transcriptome, GSEA

## Abstract

Macrophages are the foremost controllers of innate and acquired immunity, playing important roles in tissue homeostasis, vasculogenesis, and congenital metabolism. In vitro macrophages are crucial models for understanding the regulatory mechanism of immune responses and the diagnosis or treatment of a variety of diseases. Pigs are the most important agricultural animals and valuable animal models for preclinical studies, but there is no unified method for porcine macrophage isolation and differentiation at present; no systematic study has compared porcine macrophages obtained by different methods. In the current study, we obtained two M1 macrophages (M1_IFNγ + LPS, and M1_GM-CSF) and two M2 macrophages (M2_IL4 + IL10, and M2_M-CSF), and compared the transcriptomic profiles between and within macrophage phenotypes. We observed the transcriptional differences either between or within phenotypes. Porcine M1 and M2 macrophages have consistent gene signatures with human and mouse macrophage phenotypes, respectively. Moreover, we performed GSEA analysis to attribute the prognostic value of our macrophage signatures in discriminating various pathogen infections. Our study provided a framework to guide the interrogation of macrophage phenotypes in the context of health and disease. The approach described here could be used to propose new biomarkers for diagnosis in diverse clinical settings including porcine reproductive and respiratory syndrome virus (PRRSV), African swine fever virus (ASFV), *Toxoplasma gondii* (*T. gondii*), porcine circovirus type 2 (PCV2), Haemophilus parasuis serovar 4 (HPS4), *Mycoplasma hyopneumoniae* (Mhp), Streptococcus suis serotype 2 (SS2), and LPS from *Salmonella enterica* serotype minnesota Re *595*.

## 1. Introduction

Macrophages are important effectors in specific and non-specific immunity, functioning in the generation and defense of many diseases. Macrophages are derived from macrophage precursor cells and with high plasticity, including two main subtypes, M1 and M2 [1]. Macrophages exist in a variety of physiological and pathological processes, and the proportion of M1 and M2 dynamically changes [2,3,4]. M1 macrophages feature in the following aspects, producing pro-inflammatory cytokines, mediating resistance to pathogens, exhibiting strong microbicidal properties, and also contributing to tissue destruction [5,6,7,8]. Classically, M1 macrophages can be activated when cells receive stimuli such as IFNγ, LPS, as well as GM-CSF [9]. Phenotypically, M1 macrophages express high levels of MHC-II, CD68, CD80, and CD86, they are also characterized by an elevated ability to secrete cytokines such as IL1β, TNF, IL12, and IL18 [6,10,11]. In contrast, M2 macrophages are activated through a pathway opposite to the classical pathway, which responds to stimuli factors such as CSF-1, IL4, IL10, TGF-β, and IL13. M2 macrophages play a central role in responses to parasites, tissue remodeling, angiogenesis, and allergic diseases [4,12,13]. The polarization of M2 can hydrolyze L-ornithine to arginine 1 (ARG1), which is the basic amino acid that makes up proline and hydroxyproline [14]. Proline and hydroxyproline are essential amino acids of collagen, which is an important protein in tissue repair, which helps to form an external matrix related to tissue repair [15,16,17].

Owing to their crucial role in host immunity, in vitro macrophage models have been widely applied in basic research studies. Porcine macrophages are similar to human macrophages in that they have a wide range of pattern recognition receptors that detect pathogen-associated molecular patterns (PAMP) on pathogens [18]. The porcine biomedical model is ideal for many studies on human infection, inflammation, energy metabolism, and obesity [12,16,19,20,21,22]. In specific aspects, porcine is closer to human, compared to mice to human for example, the overlap degree of immune parameters of porcine and human is greater than 80% [21,23,24,25,26,27]. However, there is no standard in vitro porcine macrophage model and the differences between macrophages induced by different methods are not clear. Here, we questioned whether cultured macrophages help define specific functional phenotypes encountered in disease and the reliability of isolation, differentiation, and culture of porcine macrophages. With this in mind, a reliable method for describing the isolation, differentiation, and culture of porcine bone marrow-derived macrophages (BMDM) can be regarded as a valuable tool for classifying and studying the defined subset of macrophages found in specific environments.

Since different macrophage phenotypes are profoundly involved in the development and outcome of many microbial infected diseases, and are key cells in controlling normal physiological processes, we question whether a restricted set of gene signatures could be applied to define a particular functional phenotype encountered in the context of microbial infectious diseases. One of the most useful animal models for preclinical research is the pig. Thus, we applied RNA-seq to compare the transcriptome differences of porcine macrophages induced by different methods. By analyzing the correlation between the transcriptome of macrophages induced by different methods and the transcriptome of macrophages infected by different pathogens and at different stages of infection, a theoretical reference value was provided for the diagnosis and molecular mechanisms of swine disease, such as PRRSV, ASFV, *T. gondii*, and so on.

## 2. Materials and Methods

### 2.1. Animals

Seven day old Duroc × (Landrace × Yorkshire) hybrid pigs (DLY) used in this study were obtained from the experimental farm of Sichuan Agricultural University (Ya’an Campus). The animal experiment was approved by the Experimental Animal Ethics Committee of Sichuan Agricultural University under permit number 20210167 and was performed following the guidelines for the management and use of laboratory animals. According to the IACUC guidelines, pigs were killed by bloodletting, and then the femurs were collected to separate BMDM. The femur was separated and used for further bone marrow cell isolation.

### 2.2. Cell Culture

In this study, bone marrow cells were obtained by puncture and passed through a 40 μM cell strainer (FALCON, New York, NY, USA, 352340). After erythrocytes were removed by an ACK lysate kit (Gibco, Grand Island, Now York, NY, USA, A1049201), the mononuclear cells were resuspended and cultured with DMEM/F12 (Gibco, Grand Island, Now York, USA, 11330-0320) supplemented with 10% heat-inactivated fetal bovine serum (FBS) (Gibco, Grand Island, Now York, NY, USA, 10099141C), 100 U/mL penicillin, and 100 mg/mL streptomycin (Gibco, Temecula, CA, USA, 030311B) (DMEM/F12 10% FBS) at 37 °C in 5% CO_2_ humidified air. After 4 h, the unattached cells were enriched and seeded in a new flask for macrophage differentiation by the different stimuli detailed (M1_IFNγ + LPS: 100 ng/mL IFNγ and 20 ng/mL LPS, M1_GM-CSF: 20 ng/mL GM-CSF, M2_IL4 + IL10: 10 ng/mL IL10 and 10 ng/mL IL4, and M2_M-CSF: 20 ng/mL M-CSF) (Table 1). The experiments were performed in triplicate.

### 2.3. RNA-Seq

After the induction, macrophage cells were collected and used for total RNA extraction using Trizol (Invitrogen, San Francisco, CA, USA, 15596026), and the RNA-seq libraries were constructed using the NEBNext^®^ UltraTM RNA Library Prep Kit (NEB, Ipswich, MA, USA, 7530). The paired-end RNA-seq sequencing libraries were further sequenced by the Illumina Novaseq6000 platform (PE150), yielding 151Gb raw data and an average of 804 million 150-bp paired-end raw reads (Novogene Co. Ltd., Tianjing, China).

Sequenced reads were aligned to the pig reference genome (Sus_scrofa.Sscrofa11.1.104.gtf and Sus_scrofa.Sscrofa11.1.cdna.all.fa.gz). Gene expression was quantified by using Kallistov0.44.0 [30] and obtaining read counts for each transcript. We standardize read counts to TPM (per million transcript readings) and differential gene expression analysis was performed using the Edge R package. KEGG and GO annotation were performed using the online tool Metascape and hub genes were identified by using Metascape (https://metascape.org/gp/index.html, accessed on 4 November 2022), KEGG pathway analysis, and GO biological process analysis was performed for different genes. In order to compare the protein functions of macrophages between different methods, the protein–protein interaction network (PPI) was constructed by String (https://string-db.org, accessed on 17 November 2022) and the key Hub genes were identified.

Raw and processed RNA-seq data were deposited in the NCBI GSE202115.

### 2.4. Gene Set Enrichment Analysis (GSEA) and Network Construction

To assay, whether our gene signatures (M1_IFNγ + LPS, M1_GM-CSF, M2_IL4 + IL10, and M2_M-CSF) can discriminate macrophages infected with various pathogens (Table 2), we applied GSEA to explore the correlation between our macrophage signatures with pathogen-infected macrophages obtained from the publicly available Gene Expression Omnibus (GEO) NCBI database (http://www.ncbi.nlm.nih.gov/geo/, accessed on 30 November 2022) [31].

Since the GSEA method was originally developed for analyzing microarray data, we normalized the raw count for standard GSEA by TPM and transformed TPM into a GCT format gene expression data set. Genes were ranked based on the correlation between their expression and class distinction, by evaluating if an a priori-defined set of genes (M1_IFNγ + LPS, M1_GM-CSF, M2_IL4 + IL10, and M2_M-CSF) were randomly distributed or were primarily associated with a tested class.

## 3. Results

### 3.1. Comparing M1 with M2 to Generate Porcine Macrophage Gene Signatures

**Figure 1:**
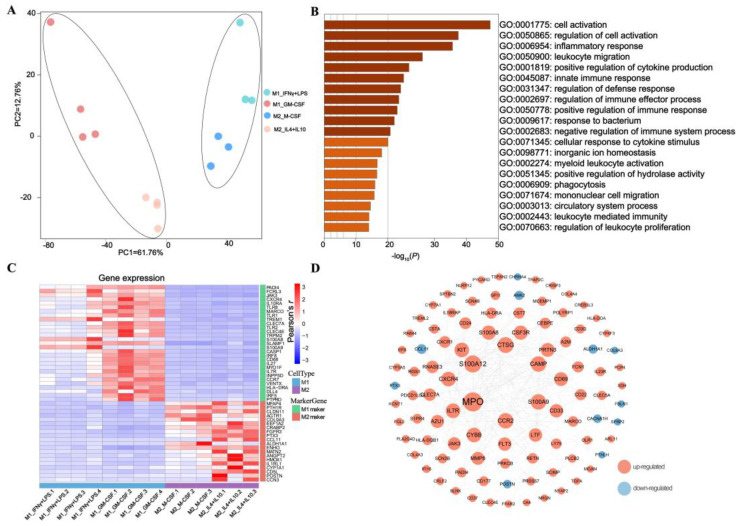
Transcriptomic comparison of porcine macrophage phenotypes, M1 (M1_IFNγ + LPS, and M1_GM-CSF) versus M2 (M1_IFNγ + LPS, and M1_GM-CSF). (**A**) PCA clustering M1_IFNγ + LPS (*n* = 4), M1_GM-CSF (*n* = 4), M2_IL4 + IL10 (*n* = 3), M2_M-CSF (*n* = 3); (**B**) Top enriched GO biological processes of 730 DEG genes, M1 (M1_IFNγ + LPS, and M1_GM-CSF) and M2 (M2_IL4 + IL10, and M2_M-CSF); (**C**) Heatmap exhibiting 49 classical macrophage marker genes expression profile among M1_IFNγ + LPS, M1_GM-CSF, M2_IL4 + IL10, and M2_M-CSF; (**D**) The DEGs between M1 and M2 were studied by using the String online tool. The interaction between each protein pair is represented by lines, and the size of the circle is directly proportional to the degree.

To fully characterize the specificity of two macrophage phenotypes polarized by different methods, we applied RNA-seq and compared the transcriptomic commonalities and differences across phenotypes and methods within phenotype. The principal components analysis (PCA) plot indicated that the macrophages clearly separated between phenotypes and within phenotype (Figure 1A). DEGs were evaluated by GO and KEGG after being transformed into gene IDs. The BP (biological process) components of the GO annotations of DEGs were used to examine the functional enrichment of DEGs. To ascertain the connection between DEGs and signaling pathways, KEGG analysis was performed. The DEGs of M1 (M1 IFN + LPS, and M1 GM-CSF) compared to M2 (M2 IL4 + IL10, and M2 M-CSF) were found to be enriched in biological processes related to immune response, such as Cell activation, Regulation of cell activation, Inflammatory response, Innate immune response, and pathways such as Hematopoietic cell lineage, Cytokine–cytokine receptor interaction (Figure 1B, Supplementary Figure S2 and Additional file S1). Moreover, we observed that the upregulated DEGs mainly enriched into phagosome pathways, pathways in cancer, and disease-related pathways, while the down-regulated genes enriched into tissue development pathways. To verify the reliability of polarized macrophages, 49 classical macrophage marker genes were retrieved from the previous literature, containing 29 and 20 macrophage markers for M1 and M2, respectively (Supplementary Table S2). Consistently, all 29 classical M1 and 20 M2 markers are highly expressed in M1 and M2, respectively (Figure 1C, Additional file S1). Using a *p* value < 0.01 and absolute log2FC value > 1 as criteria, 730 differential expressed genes (DEGs) were identified. Comparing M1 to M2 620 and 110 genes were significantly upregulated and downregulated, respectively (Additional file S1). To examine more closely the correlations between the genes of the core response to M1 macrophages (M1_IFNγ + LPS, and M1_GM-CSF) and M2 (M2_IL4 + IL10, and M2_M-CSF) macrophages, we ran a protein–protein interaction analysis using String to identify the hub genes. Ranked by connectivity and betweenness centrality, *MPO*, *S100A12*, *CTSG*, *CCR2*, *CAMP*, *S100A9*, *KIT*, *CXCR4*, and *CYBB* were identified as the major hub genes (Figure 1D, and Additional file S1).

### 3.2. Revealing Transcriptomic Differences of Macrophages within Phenotypes

**Figure 2:**
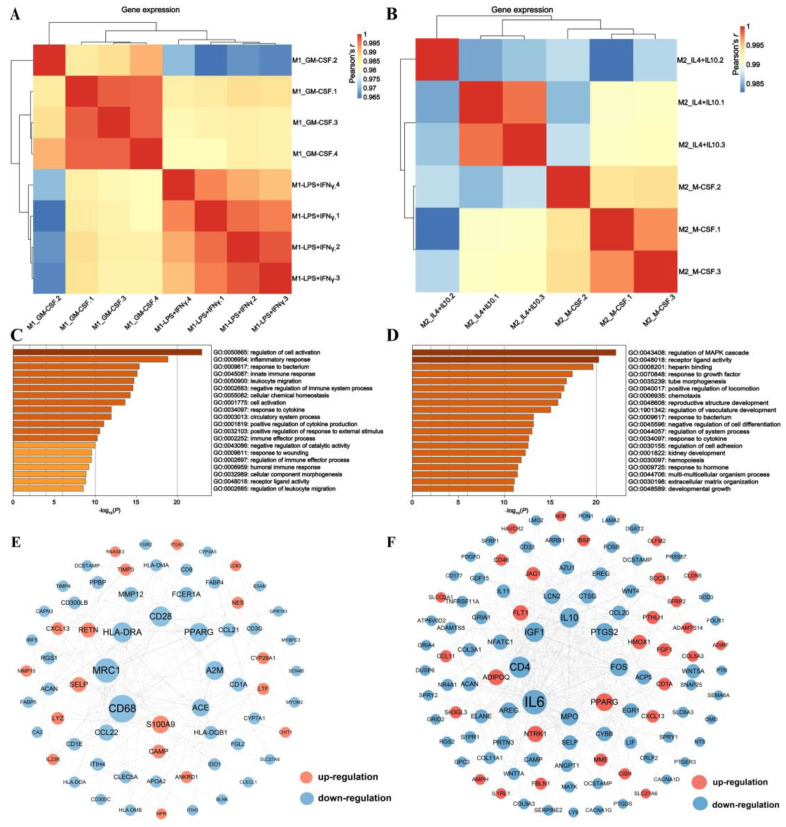
Comparison of transcriptomics in different phenotypes of porcine macrophages. (**A**) Heatmap showing that specific macrophage phenotypes were aggregated by the induction method, M1_IFNγ + LPS, and M1_GM-CSF; (**B**) Heatmap showing that specific macrophage phenotypes were aggregated by the induction method M2_IL4 + IL10 and M2_M-CSF; (**C**) GO pathway enrichment analysis of two polarized macrophages, M1_IFNγ + LPS and M1_GM-CSF; (**D**) GO pathway enrichment analysis of two polarized macrophages, M2_IL4 + IL10 and M2_M-CSF; (**E**) The DEGs were studied by using the String online tool, M1_IFNγ + LPS, and M1_GM-CSF. The interaction between each protein pair is represented by lines, and the size of the circle is directly proportional to the degree of interaction. We adopt a node number greater than 1; (**F**) The DEGs are studied by using the String online tool, M2_IL4 + IL10, and M2_M-CSF. The interaction between each protein pair is represented by lines, and the size of the circle is directly proportional to the degree of interaction. We adopt a node number greater than 2.

We also observed that the specific macrophage phenotypes were separately clustered by induction methods, M1_IFNγ + LPS versus M1_GM-CSF and M2_IL4 + IL10 versus M2_M-CSF (Figure 2). Comparing M1_IFNγ + LPS to M1_GM-CSF, 207 and 391 genes were significantly upregulated and downregulated, respectively (Additional file S2). DEGs’ annotation indicates that they are enriched in biological processes related to immune response, such as Regulation of cell activation, Inflammatory response, Response to the bacterium, and pathways such as Staphylococcus aureus infection, Cytokine–cytokine receptor interaction, Pathways in cancer (Figure 2C, Supplementary Figure S2, and Additional file S2). To examine more closely the correlations between the genes of the core response to M1_IFNγ + LPS and M1_GM-CSF, we incorporated a network-based protein–protein interaction analysis approach by String. Ranked by connectivity and betweenness centrality, *CD68*, *MRC1*, *CD28*, *HLA-DRA*, *PPARG*, *A2M*, *S100A9*, *SELP*, *RETN*, and *CAMP* were identified as the primary hub and bottleneck genes of macrophages (Figure 2E, and Additional file S2).

Comparing M2_IL4 + IL10 to M2_M-CSF, 391 and 211 genes were significantly upregulated and downregulated, respectively (Additional file S3). Running GO and KEGG annotations, the DEGs enriched in tissue development-related biological processes such as Tube morphogenesis, Reproductive structure development, Regulation of vasculature development, and pathways such as Osteoclast differentiation, Hematopoietic cell lineage, Cell adhesion molecules (Figure 2D, Supplementary Figure S3 and Additional file S3). *IL6*, *CD4*, *IGF1*, *IL10*, *PTGS2*, *FOS, PPARG*, *NTRK1*, *ADIPOQ*, *FLT1*, and *HMOX1* were identified as the primary hub and bottleneck genes of macrophages induced by colony factors and cytokines’ exposure (Figure 2F and Additional file S3).

### 3.3. Application of M1_IFNγ + LPS, M1_GM-CSF, M2_IL4 + IL10, and M2_M-CSF Signatures to the Identification of Swine Disease and Its Molecular Mechanism

As macrophages play a key role in determining the activation or resolution of immune responses and can determine the fate of pathogen infection, we evaluated the robustness of our macrophage signatures (M1_IFNγ + LPS, M1_GM-CSF, M2_IL4 + IL10, M2_M-CSF) in discriminating macrophages infected with specific pathogens based on the enrichment analysis of selected genes. The association between the chosen clinical trials where infected pig macrophages were examined in 308 transcriptomes’ data obtained from the GEO (Table 2 and Additional file S4).

**Figure 3:**
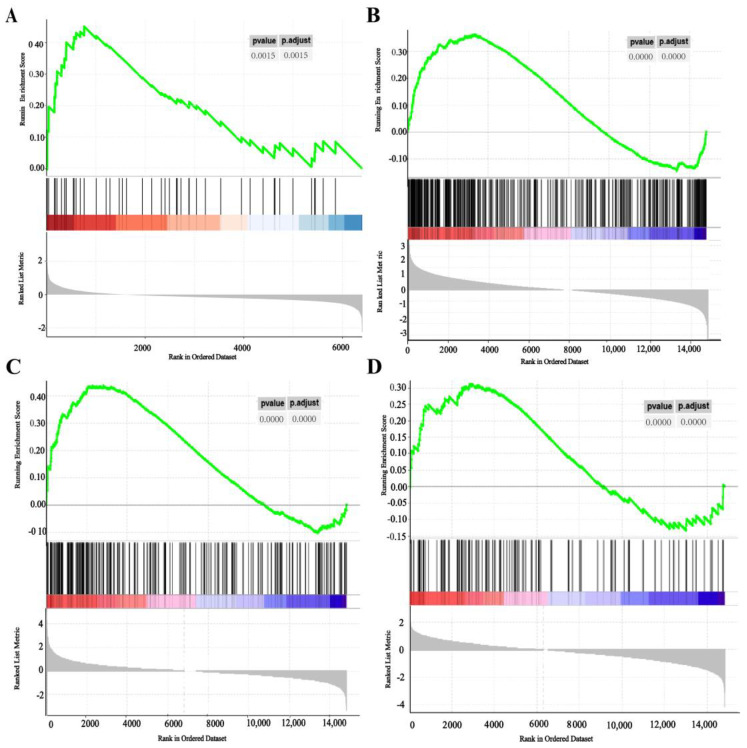
Validation of M1_IFNγ + LPS, M1_GMCSF, M2_M-CSF, and M2_IL4 + IL10 signatures. (**A**) SS2-infected macrophages were enriched in M1_IFNγ + LPS based on GSEA (Data source: Microarray analysis); (**B**) PRRSV-infected macrophages were enriched in M1_GM-CSF based on GSEA (Data source: RNA-seq); (**C**). *T. gondii*-infected macrophages were enriched in M2_IL4 + IL10 based on GSEA (Data source: RNA-seq); (**D**) *T. gondii Me49*-infected macrophages were enriched in M2_M-CSF based on GSEA (Data source: RNA-seq).

The results indicated that genes from the SS2 infected macrophages were most significantly enriched in the M1_IFNγ + LPS set, genes from the PPRSV, ASFV, Mhp, and LPS infected were most significantly enriched in the M1_GM-CSF, genes from the PCV2 and *T. gondii Me49* infections were most significantly enriched in the M2_M-CSF set, and genes from the different species of *T. gondii* infections were most significantly enriched in the M2_IL4 + IL10 set (Figure 3).

Together, our results indicate that our macrophage signatures could characterize microarrays and RNA-seq from specific pathological scenarios.

## 4. Discussion

Growing evidence shows macrophages have high plasticity and extensive polarization, which hinders the definition of macrophages obtained by different methods. The establishment of the porcine macrophage model is important for pig health and disease research. Transcriptomics is one of the primary tools in this investigation, however, we are aware of its shortcomings. Since the transcriptome is type-specific and changes over time and in response to stimulation, and some tissue-specific disorders cannot be diagnosed by RNA-seq, several studies have demonstrated that not all genes are expressed solely in particular cells [32]. Of course, considering the operability of the laboratory and the better differentiation effect of young piglets, we only used the 7-day-old DLY, which has certain limitations. In the present study, we generated four porcine macrophage phenotypes, namely M1_IFNγ + LPS, M1_GM-CSF, M2_IL4 + IL10, and M2_M-CSF and created four macrophage molecular signatures by transcriptomic analysis. First, we identified 730 DEGs by comparing M1 (merging M1_IFNγ + LPS and M1_GM-CSF) with M2 (mering M2_IL4 + IL10 and M2_M-CSF). In line with previous reports, the porcine macrophages had similar gene profiles to human and mouse classical macrophage phenotypes and alternative phenotypes, respectively. For example, porcine M1 highly expressed *CXCR4*, *S100A9*, *FCRL3*, *JAK3*, and *SLAMF1*, while M2 highly expressed *CCL11*, *POSTN*, *PTX3 MFAP4*, *PTH1R*, and *AGTR1*. Apart from the well-known macrophage markers, we noticed that porcine M1 highly expressed *MPO*, *S100A12*, *CTSG*, *CCR2*, *CAMP*, *KIT*, and *CYBB*, and M2 highly expressed *ANK2*, *ALDH1A1*, *PTHLH*, and *CACNA1H*. *MPO* is a marker of macrophage aggregation in inflammatory species and promotes the recognition of macrophage scavenger receptors [33]. *S100A12*, *CSTG*, *CAMP*, and *KIT* are antimicrobial response genes. CSTG, *CAMP*, and *CYBB* are involved in macrophage phagocytosis [34,35,36]. Both *CCR2* and *CXCR4* are pro-inflammatory genes involved in macrophage migration [37]. ANK2 promotes the growth and invasion of pancreatic carcinoma, and the peptide derived from ANK2 is an effective and specific autophagy inhibitor binding to ATG8 [38,39]. Studies have shown that the expression level of ALDH1A1 is reduced in the inflammatory state, which is part of early inflammation [40]. *PTHLH* is involved in cell and organ growth, development, migration, and survival, and can be used as an independent marker of prognosis [41]. These genes may serve as specific porcine macrophage markers and potential therapeutic targets. It is worth mentioning that four MHC haplotype genes were detected expressing in porcine bone marrow-derived macrophages, such as CIITA, HLA-DOB, HLA-DRA, SLA-DMB, and two of them, HLA-DRA and SLA-DMB, are differentially expressed, comparing M1 versus M2 [42].

After revealing the transcriptomic difference between the two main macrophage phenotypes, we conducted a transcriptomic comparison of M1_IFNγ + LPS with M1_GM-CSF, and M2_IL4 + IL10 with M2_M-CSF. We found that porcine M1_IFNγ + LPS highly expressed *S100A9*, *SELP*, *RETN*, *CAMP*, and M1_GM-CSF highly expressed *CD68*, *MRC1*, *CD28*, *HLA-DRA*, *PPARG*, and *A2M.* S100A9 is related to the CD68 regulating macrophage function pathway and promoting macrophage migration, which can induce neutrophil chemotaxis and promote macrophage migration and adhesion under inflammatory conditions [43]. RETN has been reported to induce the production of pro-inflammatory cytokines and chemokines in PBMC [44]. CAMP can inhibit the phagocytosis of macrophages through the CAMP-1-activated CAMP-effect-exchange protein [45]. *CD68* is highly expressed in macrophages and belongs to the scavenger family. It has the functions of clearing cell debris, promoting phagocytosis, and mediating the recruitment and activation of macrophages. MRC1 and A2M mediate the phagocytosis of macrophages [43]. CD28 can enhance the expression of RANTES and MIP-1α in T cells and MIP-1β, and increase the number and differentiation of macrophages in the wound healing stage [9,46]. HLA-DRA has been proven to inhibit the phagocytosis of macrophages in order to protect the intracellular niche from phagocytosis and killing of host macrophages, which is positively related to the regulation of GM-CSF [47,48,49].

We found that porcine M2_IL4 + IL10 highly expressed *PPARG*, *NTRK1*, *ADIPOQ*, *FLT1*, *HMOX1*, and M2_M-CSF highly expressed *IL6*, *CD4*, *IGF1*, *IL10*, *PTGS2*, *FOS*, *PPARG* [50,51,52]. PPARG has an anti-inflammatory effect, and its ligand is responsible for clearing the expression of genes of apoptotic cells and macrophage-mediated inflammatory responses [53,54]. Interestingly, PPARG was also specifically expressed in M1_GM-CSF. NTRK1 decreases malignancy and/or spontaneous degeneration of neuroblastoma cells [55]. ADIPOQ and FLT1 have anti-inflammatory functions [56,57,58]. According to reports, HMOX1 also has anti-cancer, anti-inflammatory, anti-apoptotic, anti-proliferation, and anti-oxidation effects [59]. Although IL6 is usually associated with pro-inflammatory function and involves many inflammatory diseases, it can increase the polarization of alternatively activated (AAM) [60]. AAM and IL6 together, lead to the release of IL10 [60,61]. At the same time, FOS can increase the expression level of IL10 and promote the formation of osteoclasts [62]. IGF1 and IL10 are also positively correlated with the inhibition of inflammation and wound healing. Interestingly, IL10 also promotes macrophage phagocyte debris [63]. CD4 has an anti-inflammatory effect, inhibits macrophage migration, and induces M2 polarization [64]. Interestingly, *PTGS2* is a pro-inflammatory gene [65].

We have investigated the variations among various phenotypes and associated them with disease outcomes because we are interested in investigating the potential application of macrophage phenotypes. We proposed a consensus collection of markers describing major macrophages’ activation phenotypes, namely M1_IFNγ + LPS, M1_GM-CSF, M2_IL4 + IL10, and M2_M-CSF, able to characterize robustly controlled in vitro and in vivo scenarios for macrophage induction. Our study confirmed that macrophages cultured in vitro have different enrichment pathways from macrophages infected with different pathogens. At the same time, it is confirmed that macrophages infected with the same pathogen in vivo or in vitro also have different enrichment pathways. Since the description of the polarization state of macrophages has not been unified, our study provides a framework to guide the definition of the phenotype of porcine macrophages in the disease state. Future research into the macrophage model in a disease setting will help us create medications and diagnostic tools for particular disorders. Nevertheless, we can never ignore the bias using public downloaded transcriptomic data.

We conclude here that there are transcriptomic differences between and within two macrophage phenotypes in our porcine model. In general, we found the four types of macrophages (M1_IFNγ + LPS, M1_GM-CSF, M2_IL4 + IL10, and M2_M-CSF) have different functions. Compared with M1_GM-CSF, M1_IFNγ + LPS has a weaker phagocytic capacity, but stronger antibacterial and migratory capacity; M2_IL4 + IL10 has a stronger tissue repair function, while M2_M-CSF has a stronger wound healing ability [66]. At the same time, we used the four established gene characteristics to identify various pig infectious diseases with prognosis and predictive values. Pigs’ immunological parameters overlap with either mice or humans’ in particular ways more than mice and humans’ do with each other [21,23,24,25,26,27]. Indeed, it would be an interesting research direction to establish similar models of pig, mouse, and human macrophages for future studies.

## Figures and Tables

**Table 1:** Brief methods of bone marrow-derived macrophage induction.

Abbreviation	Phenotype	Method
M1_IFNγ + LPS	M1	100 ng/mL IFNγ (Proteintech, Rosemount, IL, USA, HZ-1301), and 20 ng/mL LPS (sigma, USA, L4516)
M1_GM-CSF	M1	20 ng/mL GM-CSF (absin, Shanghai, China, abs04132)
M2_IL4 + IL10	M2	10 ng/mL IL10(Proteintech, USA, HZ-1145), and 10 ng/mL IL4 (Proteintech, Rosemount, IL, USA, HZ-1004)
M2_M-CSF	M2	20 ng/mL M-CSF (absin, Shanghai, China, abs00846)

Macrophage induction methods were adapted from [19,28,29].

**Table 2:** Gene Set Enrichment Analysis (GSEA) in clinical samples.

GEO ID	Cohort Description	Experimental Groups
GSE174494	Macrophages were obtained by lung perfusion in pigs infected with the PRRSV virus. The harvested infected macrophages were detected by RNA-seq.	Alveolar M ϕ from PRRSV (*n* = 3) vs. PPRRSV-GFP (*n* = 3) vs. RPMI-1640 (*n* = 3).
GSE145954	PAMs infected ASFV isolates (MOI = 1), and transcriptome analysis was performed on infected cells and normal PAM at 0, 6, 12, and 24 h.	Alveolar M ϕ from ASFV-0h (*n* = 3) vs. ASFV-6h (*n* = 3) vs. ASFV-12h (*n* = 3) vs. ASFV-24 h (*n* = 3) vs. uninfected (*n* = 3).
GSE146715	PAM-infected with *T. gondii Me49* for 15 h. Transcriptome analysis was performed on infected and uninfected PAM.	Alveolar M ϕ from *T. gondii Me49*-PAM (*n* = 3) vs. uninfected (*n* = 3).
GSE153330	PAM-infected with TgRH (type i), TgME49 (type ii), or TgHB1-Toxoplasma for 6, 12, and 24 h. Transcriptome analysis was performed on infected and uninfected PAM.	Alveolar M ϕ from PAM at different infection times for each infection strain (*n* = 3) vs. uninfected (*n* = 3).
GSE34544	The piglets were infected with SS2 and HPS4 28 days later. Transcriptome analysis of infected and uninfected PAM.	Alveolar M ϕ from PAM for each infection strain (*n* = 6) vs. uninfected (*n* = 6).
GSE30696		
GSE181105	Infected PAM were obtained 6 and 15 h after infection with Mhp or PRRSV. Microarray analysis of infected and uninfected PAM.	Alveolar M ϕ from PAM for each infection strain (*n* = 3) vs. uninfected (*n* = 3).
GSE22782	Infected PAM were obtained after piglets were infected with HP-PRRSV for 5 days. Microarray analysis of infected and uninfected PAM.	Alveolar M ϕ from PAM for each infection strain (*n* = 6) vs. uninfected (*n* = 6).
GSE30918	Infected PAM were obtained 48 h after PCV2 infection. Microarray analysis of infected and uninfected PAM.	Alveolar M ϕ from PAM for each infection strain (*n* = 3) vs. uninfected (*n* = 3).
GSE156504	Infected PAM were obtained after 21 h of infection with different strains of PRRSV. Transcriptome analysis of infected and uninfected PAM.	Alveolar M ϕ from PAM for each infection strain (*n* = 3) vs. uninfected (*n* = 3).
GSE49882	Infected PAM were obtained after 6 and 15 h of infection with *Mycoplasma hyopneumoniae* and PRRSV.	Alveolar M ϕ from PAM for each infection strain (*n* = 3) vs. uninfected (*n* = 3).
GSE30956	BMDM were stimulated with LPS from *Salmonella enterica* serotype Minnesota Re 595 for 0, 2, 7, and 24 h to obtain macrophages after a reaction.	Alveolar M ϕ from PAM for each infection strain (*n* = 3) vs. uninfected (*n* = 3).
GSE45145	BMDM from different breeds of pigs were stimulated with LPS from *Salmonella enterica* serotype Minnesota Re 595 for 0, 2, 7, and 24 h to obtain macrophages after the reaction.	Alveolar M ϕ from PAM for each infection strain (*n* = 3) vs. uninfected (*n* = 3).

## Data Availability

Data is contained within the article.

## Supplementary Materials

The following supporting information can be downloaded at: https://www.mdpi.com/article/10.3390/cimb45030151/s1, Figure S1: The KEGG or GO Analysis of M1 and M2; Figure S2: The KEGG or GO Analysis of M1_IFNγ + LPS and M1_GM-CSF; Figure S3: The KEGG or GO Analysis of M2_IL4 + IL10 and M2_M-CSF; Table S1: Data output quality list. Table S2: The observed marker genes of M1 and M2 macrophages. References [67,68,69,70,71,72,73,74,75,76,77,78,79,80,81,82,83,84,85,86,87,88,89,90,91,92,93,94,95,96,97,98,99,100,101,102,103,104,105,106,107,108,109] are cited in the supplementary materials.

## References

[1] Takahashi K. (2001). Development and Differentiation of Macrophages and Related Cells: Historical Review and Current Concepts. J. Clin. Exp. Hematop..

[2] Murray P.J., Wynn T.A. (2011). Protective and Pathogenic Functions of Macrophage Subsets. Nat. Rev. Immunol..

[3] Sica A., Mantovani A. (2012). Plasticity and Polarization. J. Clin. Investig..

[4] Ma W., Gao F., Gu K., Chen D. (2019). The Role of Monocytes and Macrophages in Autoimmune Diseases: A Comprehensive Review. Front. Immunol..

[5] Rendra E., Riabov V., Mossel D.M., Sevastyanova T., Harmsen M.C., Kzhyshkowska J. (2019). Reactive Oxygen Species (ROS) in Macrophage Activation and Function in Diabetes. Immunobiology.

[6] Martinez F.O., Gordon S. (2014). The M1 and M2 Paradigm of Macrophage Activation: Time for Reassessment. F1000Prime Rep..

[7] Bility M.T., Cheng L., Zhang Z., Luan Y., Li F., Chi L., Zhang L., Tu Z., Gao Y., Fu Y.-X. (2014). Hepatitis B Virus Infection and Immunopathogenesis in a Humanized Mouse Model: Induction of Human-Specific Liver Fibrosis and M2-Like Macrophages. PLoS Pathog..

[8] Anderson N.R., Minutolo N.G., Gill S., Klichinsky M. (2021). Macrophage-Based Approaches for Cancer Immunotherapy. Cancer Res..

[9] Lacey D.C., Achuthan A., Fleetwood A.J., Dinh H., Roiniotis J., Scholz G.M., Chang M.W., Beckman S.K., Cook A.D., Hamilton J.A. (2012). Defining GM-CSF– and Macrophage-CSF–Dependent Macrophage Responses by In Vitro Models. J. Immunol..

[10] Martinez F.O., Gordon S., Locati M., Mantovani A. (2006). Transcriptional Profiling of the Human Monocyte-to-Macrophage Differentiation and Polarization: New Molecules and Patterns of Gene Expression. J. Immunol..

[11] Vogel D.Y.S., Vereyken E.J.F., Glim J.E., Heijnen P.D.A.M., Moeton M., van der Valk P., Amor S., Teunissen C.E., van Horssen J., Dijkstra C.D. (2013). Macrophages in Inflammatory Multiple Sclerosis Lesions Have an Intermediate Activation Status. J. Neuroinflamm..

[12] Swindle M.M., Makin A., Herron A.J., Clubb F.J., Frazier K.S. (2012). Swine as Models in Biomedical Research and Toxicology Testing. Vet. Pathol..

[13] Sironi M. (2006). Differential Regulation of Chemokine Production by Fc Receptor Engagement in Human Monocytes: Association of CCL1 with a Distinct Form of M2 Monocyte Activation (M2b, Type 2). J. Leukoc. Biol..

[14] Mantovani A., Sozzani S., Locati M., Allavena P., Sica A. (2002). Macrophage Polarization: Tumor-Associated Macrophages as a Paradigm for Polarized M2 Mononuclear Phagocytes. Trends Immunol..

[15] Morris S.M. (2007). Arginine Metabolism: Boundaries of Our Knowledge. J. Nutr..

[16] Perleberg C., Kind A., Schnieke A. (2018). Genetically Engineered Pigs as Models for Human Disease. DMM Dis. Model. Mech..

[17] Pepe G., Calderazzi G., De Maglie M., Villa A.M., Vegeto E. (2014). Heterogeneous Induction of Microglia M2a Phenotype by Central Administration of Interleukin-4. J. Neuroinflamm..

[18] Giraud E., Lestinova T., Derrick T., Martin O., Dillon R.J., Volf P., Műller I., Bates P.A., Rogers M.E. (2018). Leishmania Proteophosphoglycans Regurgitated from Infected Sand Flies Accelerate Dermal Wound Repair and Exacerbate Leishmaniasis via Insulin-like Growth Factor 1-Dependent Signalling. PLoS Pathog..

[19] Leidi M., Gotti E., Bologna L., Miranda E., Rimoldi M., Sica A., Roncalli M., Palumbo G.A., Introna M., Golay J. (2009). M2 Macrophages Phagocytose Rituximab-Opsonized Leukemic Targets More Efficiently than M1 Cells In Vitro. J. Immunol..

[20] Harusato A., Geem D., Denning T.L. (2016). Macrophage Isolation from the Mouse Small and Large Intestine. Methods Mol. Biol..

[21] Truty M.J., Smoot R.L. (2008). Animal Models in Pancreatic Surgery: A Plea for Pork. Pancreatology.

[22] Martinez F.O., Helming L., Milde R., Varin A., Melgert B.N., Draijer C., Thomas B., Fabbri M., Crawshaw A., Ho L.P. (2013). Genetic Programs Expressed in Resting and IL-4 Alternatively Activated Mouse and Human Macrophages: Similarities and Differences. Blood J. Am. Soc. Hematol..

[23] Gao J., Scheenstra M.R., van Dijk A., Veldhuizen E.J.A., Haagsman H.P. (2018). A New and Efficient Culture Method for Porcine Bone Marrow-Derived M1-and M2-Polarized Macrophages. Vet. Immunol. Immunopathol..

[24] Raes G., Van den Bergh R., De Baetselier P., Ghassabeh G.H. (2005). Arginase-1 and Ym1 Are Markers for Murine, but Not Human, Alternatively Activated Myeloid Cells. J. Immunol..

[25] Schneemann M., Schoeden G. (2007). Macrophage Biology and Immunology: Man Is Not a Mouse. J. Leukoc. Biol..

[26] Meurens F., Summerfield A., Nauwynck H., Saif L., Gerdts V. (2012). The Pig: A Model for Human Infectious Diseases. Trends Microbiol..

[27] Mehle A., Doudna J.A. (2009). Adaptive Strategies of the Influenza Virus Polymerase for Replication in Humans. Proc. Natl. Acad. Sci. USA.

[28] Mclaughlin T., Ackerman S.E., Shen L., Engleman E. (2017). Role of Innate and Adaptive Immunity in Obesity-Associated Metabolic Disease. J. Clin. Investig..

[29] Björck P. (2001). Isolation and Characterization of Plasmacytoid Dendritic Cells from Flt3 Ligand and Granulocyte-Macrophage Colony-Stimulating Factor-Treated Mice. Blood.

[30] Bray N.L., Pimentel H., Melsted P., Pachter L. (2016). Near-Optimal Probabilistic RNA-Seq Quantification. Nat. Biotechnol..

[31] Subramanian A., Tamayo P., Mootha V.K., Mukherjee S., Ebert B.L., Gillette M.A., Paulovich A., Pomeroy S.L., Golub T.R., Lander E.S. (2005). Gene Set Enrichment Analysis: A Knowledge-Based Approach for Interpreting Genome-Wide Expression Profiles. Proc. Natl. Acad. Sci. USA.

[32] Cirulli E.T., Singh A., Shianna K.V., Ge D., Smith J.P., Maia J.M., Heinzen E.L., Goedert J.J., Goldstein D.B. (2010). Screening the Human Exome: A Comparison of Whole Genome and Whole Transcriptome Sequencing. Genome Biol..

[33] Luo H., Ma C. (2021). A Novel Ferroptosis-Associated Gene Signature to Predict Prognosis in Patients with Uveal Melanoma. Diagnostics.

[34] Li X., Yu W., Wollenweber T., Lu X., Wei Y., Beitzke D., Wadsak W., Kropf S., Wester H.J., Haug A.R. (2019). [68 Ga] Pentixafor PET/MR Imaging of Chemokine Receptor 4 Expression in the Human Carotid Artery. Eur. J. Nucl. Med. Mol. Imaging.

[35] Tang C., Lei X., Xiong L., Hu Z., Tang B. (2021). HMGA1B/2 Transcriptionally Activated-POU1F1 Facilitates Gastric Carcinoma Metastasis via CXCL12/CXCR4 Axis-Mediated Macrophage Polarization. Cell Death Dis..

[36] Emokpae M.A., Mrakpor B.A. (2016). Do Sex Differences in Respiratory Burst Enzyme Activities Exist in Human Immunodeficiency Virus-1 Infection?. Med. Sci..

[37] Wu W., Hou B., Tang C., Liu F., Yang J., Pan T., Si K., Lu D., Wang X., Wang J. (2018). (+)-Usnic Acid Inhibits Migration of c-KIT Positive Cells in Human Colorectal Cancer. Evid.-Based Complement. Altern. Med..

[38] Van Rossum D., Hilbert S., Straßenburg S., Hanisch U., Brück W. (2008). Myelin-phagocytosing Macrophages in Isolated Sciatic and Optic Nerves Reveal a Unique Reactive Phenotype. Glia.

[39] Cao W., Wei W., Zhan Z., Xie D., Xie Y., Xiao Q. (2018). Regulation of Drug Resistance and Metastasis of Gastric Cancer Cells via the MicroRNA647-ANK2 Axis. Int. J. Mol. Med..

[40] Auburger G., Gispert S., Torres-Odio S., Jendrach M., Brehm N., Canet-Pons J., Key J., Sen N.-E. (2019). SerThr-PhosphoProteome of Brain from Aged PINK1-KO+ A53T-SNCA Mice Reveals PT1928-MAP1B and PS3781-ANK2 Deficits, as Hub between Autophagy and Synapse Changes. Int. J. Mol. Sci..

[41] Wu X., Lv D., Cai C., Zhao Z., Wang M., Chen W., Liu Y. (2020). A TP53-Associated Immune Prognostic Signature for the Prediction of Overall Survival and Therapeutic Responses in Muscle-Invasive Bladder Cancer. Front. Immunol..

[42] Foss D.L., Bennaars A.M., Pennell C.A., Moody M.D., Murtaugh M.P. (2003). Differentiation of Porcine Dendritic Cells by Granulocyte-Macrophage Colony-Stimulating Factor Expressed in Pichia Pastoris. Vet. Immunol. Immunopathol..

[43] Bézie S., Freuchet A., Sérazin C., Salama A., Vimond N., Anegon I., Guillonneau C. (2020). IL-34 Actions on FOXP3+ Tregs and CD14+ Monocytes Control Human Graft Rejection. Front. Immunol..

[44] Mossel D.M., Moganti K., Riabov V., Weiss C., Kopf S., Cordero J., Dobreva G., Rots M.G., Klüter H., Harmsen M.C. (2020). Epigenetic Regulation of S100A9 and S100A12 Expression in Monocyte-Macrophage System in Hyperglycemic Conditions. Front. Immunol..

[45] Pan L., Wei N., Jia H., Gao M., Chen X., Wei R., Sun Q., Gu S., Du B., Xing A. (2017). Genome-Wide Transcriptional Profiling Identifies Potential Signatures in Discriminating Active Tuberculosis from Latent Infection. Oncotarget.

[46] Carroll R.G., Riley J.L., Levine B.L., Feng Y., Kaushal S., Ritchey D.W., Bernstein W., Weislow O.S., Brown C.R., Berger E.A. (1997). Differential Regulation of HIV-1 Fusion Cofactor Expression by CD28 Costimulation of CD4+ T Cells. Science.

[47] Gladow N., Hollmann C., Ramos G., Frantz S., Kerkau T., Beyersdorf N., Hofmann U. (2020). Treatment of Mice with a Ligand Binding Blocking Anti-CD28 Monoclonal Antibody Improves Healing after Myocardial Infarction. PLoS ONE.

[48] Vozza E.G., Mulcahy M.E., McLoughlin R.M. (2021). Making the Most of the Host; Targeting the Autophagy Pathway Facilitates Staphylococcus Aureus Intracellular Survival in Neutrophils. Front. Immunol..

[49] Perry S.E., Mostafa S.M., Wenstone R., Shenkin A., McLaughlin P.J. (2004). HLA-DR Regulation and the Influence of GM-CSF on Transcription, Surface Expression and Shedding. Int. J. Med. Sci..

[50] Peng P., Zhu H., Liu D., Chen Z., Zhang X., Guo Z., Dong M., Wan L., Zhang P., Liu G. (2022). TGFBI Secreted by Tumor-Associated Macrophages Promotes Glioblastoma Stem Cell-Driven Tumor Growth via Integrin Avβ5-Src-Stat3 Signaling. Theranostics.

[51] Pasqualetti F., Giampietro C., Montemurro N., Giannini N., Gadducci G., Orlandi P., Natali E., Chiarugi P., Gonnelli A., Cantarella M. (2022). Old and New Systemic Immune-Inflammation Indexes Are Associated with Overall Survival of Glioblastoma Patients Treated with Radio-Chemotherapy. Genes.

[52] Li D., Zhang Q., Li L., Chen K., Yang J., Dixit D., Gimple R.C., Ci S., Lu C., Hu L. (2022). Β2-Microglobulin Maintains Glioblastoma Stem Cells and Induces M2-like Polarization of Tumor-Associated Macrophages. Cancer Res..

[53] Vikhe P.P., Purnell T., Brown S.D.M., Hood D.W. (2019). Cellular Immune Response against Nontypeable Haemophilus Influenzae Infecting the Preinflamed Middle Ear of the Junbo Mouse. Infect. Immun..

[54] Glas J., Seiderer J., Markus C., Pfennig S., Wetzke M., Paschos E., Göke B., Ochsenkühn T., Müller-Myhsok B., Diegelmann J. (2011). Role of PPARG Gene Variants in Inflammatory Bowel Disease. Inflamm. Bowel Dis..

[55] Zahuczky G., Kristóf E., Majai G., Fésüs L. (2011). Differentiation and Glucocorticoid Regulated Apopto-Phagocytic Gene Expression Patterns in Human Macrophages. Role of Mertk in Enhanced Phagocytosis. PLoS ONE.

[56] Pajtler K.W., Rebmann V., Lindemann M., Schulte J.H., Schulte S., Stauder M., Leuschner I., Schmid K., Köhl U., Schramm A. (2013). Expression of NTRK1/TrkA Affects Immunogenicity of Neuroblastoma Cells. Int. J. Cancer.

[57] Pereira G., Bexiga R., Silva J.C.E., Silva E., Ramé C., Dupont J., Guo Y., Humblot P., Lopes-da-Costa L. (2020). Adipokines as Biomarkers of Postpartum Subclinical Endometritis in Dairy Cows. Reproduction.

[58] Otowa Y., Moriwaki K., Sano K., Shirakabe M., Yonemura S., Shibuya M., Rossant J., Suda T., Kakeji Y., Hirashima M. (2016). Flt1/VEGFR1 Heterozygosity Causes Transient Embryonic Edema. Sci. Rep..

[59] Lokki A.I., Heikkinen-Eloranta J.K., Laivuori H. (2018). The Immunogenetic Conundrum of Preeclampsia. Front. Immunol..

[60] Fernando M.R., Giembycz M.A., McKay D.M. (2016). Bidirectional Crosstalk via IL-6, PGE2 and PGD2 between Murine Myofibroblasts and Alternatively Activated Macrophages Enhances Anti-Inflammatory Phenotype in Both Cells. Br. J. Pharmacol..

[61] Keränen T., Hömmö T., Hämäläinen M., Moilanen E., Korhonen R. (2016). Anti-Inflammatory Effects of Β2-Receptor Agonists Salbutamol and Terbutaline Are Mediated by MKP-1. PLoS ONE.

[62] Barroso F.A.L., de Jesus L.C.L., de Castro C.P., Batista V.L., Ferreira Ê., Fernandes R.S., de Barros A.L.B., Leclerq S.Y., Azevedo V., Mancha-Agresti P. (2021). Intake of Lactobacillus Delbrueckii (PExu: Hsp65) Prevents the Inflammation and the Disorganization of the Intestinal Mucosa in a Mouse Model of Mucositis. Microorganisms.

[63] Bergmann C.B., Beckmann N., Salyer C.E., Hanschen M., Crisologo P.A., Caldwell C.C. (2021). Potential Targets to Mitigate Trauma-or Sepsis-Induced Immune Suppression. Front. Immunol..

[64] Fernando M.R., Reyes J.L., Iannuzzi J., Leung G., McKay D.M. (2014). The Pro-Inflammatory Cytokine, Interleukin-6, Enhances the Polarization of Alternatively Activated Macrophages. PLoS ONE.

[65] Messing M., Jan-Abu S.C., McNagny K. (2020). Group 2 Innate Lymphoid Cells: Central Players in a Recurring Theme of Repair and Regeneration. Int. J. Mol. Sci..

[66] WERNER S., GROSE R. (2003). Regulation of Wound Healing by Growth Factors and Cytokines. Physiol. Rev..

[67] Acosta J.R., Tavira B., Douagi I., Kulyté A., Arner P., Rydén M., Laurencikiene J. (2019). Human-Specific Function of IL-10 in Adipose Tissue Linked to Insulin Resistance. J. Clin. Endocrinol. Metab..

[68] Al-Rubaie A., Wise A.F., Sozo F., De Matteo R., Samuel C.S., Harding R., Ricardo S.D. (2018). The therapeutic effect of mesenchymal stem cells on pulmonary myeloid cells following neonatal hyperoxic lung injury in mice. Respir. Res..

[69] Awad F., Assrawi E., Jumeau C., Georgin-Lavialle S., Cobret L., Duquesnoy P., Piterboth W., Thomas L., Stankovic-Stojanovic K., Louvrier C. (2017). Impact of human monocyte and macrophage polarization on NLR expression and NLRP3 inflammasome activation. PLoS ONE.

[70] Beceiro S., Radin J., Chatuvedi R., Piazuelo M., Horvarth D., Cortado H., Gu Y., Dixon B., Gu C., Lange I. (2017). TRPM2 ion channels regulate macrophage polarization and gastric inflammation during Helicobacter pylori infection. Mucosal Immunol..

[71] Binnemars-Postma K., Bansal R., Storm G., Prakash J. (2018). Targeting the Stat6 pathway in tumor-associated macrophages reduces tumor growth and metastatic niche formation in breast cancer. FASEB J..

[72] Camiolo G., Barbato A., Giallongo C., Vicario N., Romano A., Parrinello N.L., Parenti R., Sandoval J.C., García-Moreno D., Lazzarino G. (2020). Iron regulates myeloma cell/macrophage interaction and drives resistance to bortezomib. Redox Biol..

[73] Chen C., Perry T.L., Chitko-McKown C.G., Smith A.D., Cheung L., Beshah E., Urban J.F., Dawson H.D. (2019). The regulatory actions of retinoic acid on M2 polarization of porcine macrophages. Dev. Comp. Immunol..

[74] Chen P.-C., Cheng H.-C., Wang J., Wang S.-W., Tai H.-C., Lin C.-W., Tang C.-H. (2014). Prostate cancer-derived CCN3 induces M2 macrophage infiltration and contributes to angiogenesis in prostate cancer microenvironment. Oncotarget.

[75] Cheng Y., Si Y., Wang L., Ding M., Yu S., Lu L., Guo Y., Zong M., Fan L. (2021). The regulation of macrophage polarization by hypoxia-PADI4 coordination in Rheumatoid arthritis. Int. Immunopharmacol..

[76] Chiba Y., Mizoguchi I., Furusawa J., Hasegawa H., Ohashi M., Xu M., Owaki T., Yoshimoto T. (2018). Interleukin-27 exerts its antitumor effects by promoting differentiation of hematopoietic stem cells to M1 macrophages. Cancer Res..

[77] Cui Y., Chen F., Gao J., Lei M., Wang D., Jin X., Guo Y., Shan L., Chen X. (2021). Comprehensive landscape of the renin-angiotensin system in Pan-cancer: A potential downstream mediated mechanism of SARS-CoV-2. Int. J. Biol. Sci..

[78] Cui Z., Feng Y., Li D., Li T., Gao P., Xu T. (2020). Activation of aryl hydrocarbon receptor (AhR) in mesenchymal stem cells modulates macrophage polarization in asthma. J. Immunotoxicol..

[79] Dong H., Xie C., Jiang Y., Li K., Lin Y., Pang X., Xiong X., Zheng J., Ke X., Chen Y. (2021). Tumor-Derived Exosomal Protein Tyrosine Phosphatase Receptor Type O Polarizes Macrophage to Suppress Breast Tumor Cell Invasion and Migration. Front. Cell Dev. Biol..

[80] Fang X.Y., Zhan Y.X., Zhou X.M., Wu L.N., Lin J., Yi Y.T., Jiang C.M., Wang J., Liu J. (2021). CXCL12/CXCR4 Mediates Orthodontic Root Resorption via Regulating the M1/M2 Ratio. J. Dent. Res..

[81] Guo Y., Lin C., Xu P., Wu S., Fu X., Xia W., Yao M. (2016). AGEs induced autophagy impairs cutaneous wound healing via stimulating macrophage polarization to M1 in diabetes. Sci. Rep..

[82] Huang Y., Li Q., Hu R., Li R., Yang Y. (2022). Five immune-related genes as diagnostic markers for endometriosis and their correlation with immune infiltration. Front. Endocrinol..

[83] Inoue T. (2017). M1 macrophage triggered by Mincle leads to a deterioration of acute kidney injury. Kidney Int..

[84] Jablonski K.A., Gaudet A.D., Amici S.A., Popovich P.G., Guerau-De-Arellano M. (2016). Control of the inflammatory macrophage transcriptional signature by miR-155. PLoS ONE.

[85] Joerink M., Rindsjö E., van Riel B., Alm J., Papadogiannakis N. (2011). Placental macrophage (Hofbauer cell) polarization is independent of maternal allergen-sensitization and presence of chorioamnionitis. Placenta.

[86] Ganta V.C., Choi M., Farber C.R., Annex B.H. (2019). Antiangiogenic VEGF165b regulates macrophage polarization via S100A8/S100A9 in peripheral artery disease. Circulation.

[87] Le Y., Gao H., Bleday R., Zhu Z. (2018). The homeobox protein VentX reverts immune suppression in the tumor microenvironment. Nat. Commun..

[88] Liu M., Li F., Bin Liu B., Jian Y., Zhang D., Zhou H., Wang Y., Xu Z. (2021). Profiles of immune cell infiltration and immune-related genes in the tumor microenvironment of esophageal squamous cell carcinoma. BMC Med. Genomics.

[89] Lv D., Wu X., Chen X., Yang S., Chen W., Wang M., Liu Y., Gu D., Zeng G. (2021). A novel immune-related gene-based prognostic signature to predict biochemical recurrence in patients with prostate cancer after radical prostatectomy. Cancer Immunol. Immunother..

[90] Müller E., Christopoulos P.F., Halder S., Lunde A., Beraki K., Speth M., Øynebråten I., Corthay A. (2017). Toll-like receptor ligands and interferon-γ synergize for induction of antitumor M1 macrophages. Front. Immunol..

[91] Oliveira L., McClellan S., Hansen P.J. (2010). Differentiation of the endometrial macrophage during pregnancy in the cow. PLoS ONE.

[92] Pagie S., Gérard N., Charreau B. (2018). Notch signaling triggered via the ligand DLL4 impedes M2 macrophage differentiation and promotes their apoptosis. Cell Commun. Signal..

[93] Quan Q., Xiong X., Wu S., Yu M. (2021). Identification of Immune-Related Key Genes in Ovarian Cancer Based on WGCNA. Front. Genet..

[94] Quero L., Tiaden A.N., Hanser E., Roux J., Laski A., Hall J., Kyburz D. (2020). miR-221-3p Drives the Shift of M2-Macrophages to a Pro-Inflammatory Function by Suppressing JAK3/STAT3 Activation. Front. Immunol..

[95] Sanjurjo L., Aran G., Téllez É., Amézaga N., Armengol C., López D., Prats C., Sarrias M.-R. (2018). CD5L promotes M2 macrophage polarization through autophagy-mediated upregulation of ID3. Front. Immunol..

[96] Sato K., Yamashita T., Shirai R., Shibata K., Okano T., Yamaguchi M., Mori Y., Hirano T., Watanabe T. (2018). Adropin contributes to anti-atherosclerosis by suppressing monocyte-endothelial cell adhesion and smooth muscle cell proliferation. Int. J. Mol. Sci..

[97] Seneviratne A., Cave L., Hyde G., Moestrup S.K., Carling D., Mason J., O Haskard D., Boyle J.J. (2021). Metformin directly suppresses atherosclerosis in normoglycaemic mice via haematopoietic adenosine monophosphate-activated protein kinase. Cardiovasc. Res..

[98] Shapouri-Moghaddam A., Mohammadian S., Vazini H., Taghadosi M., Esmaeili S.-A., Mardani F., Seifi B., Mohammadi A., Afshari J.T., Sahebkar A. (2018). Macrophage plasticity, polarization, and function in health and disease. J. Cell. Physiol..

[99] Song W.-M., Agrawal P., Von Itter R., Fontanals-Cirera B., Wang M., Zhou X., Mahal L.K., Hernando E., Zhang B. (2021). Network models of primary melanoma microenvironments identify key melanoma regulators underlying prognosis. Nat. Commun..

[100] Talamonti E., Pauter A.M., Asadi A., Fischer A.W., Chiurchiù V., Jacobsson A. (2017). Impairment of systemic DHA synthesis affects macrophage plasticity and polarization: Implications for DHA supplementation during inflammation. Cell. Mol. Life Sci..

[101] Tan Y., Sun R., Liu L., Yang D., Xiang Q., Li L., Tang J., Qiu Z., Peng W., Wang Y. (2021). Tumor suppressor DRD2 facilitates M1 macrophages and restricts NF-κB signaling to trigger pyroptosis in breast cancer. Theranostics.

[102] Bossche J.V.D., Laoui D., Morias Y., Movahedi K., Raes G., De Baetselier P., Van Ginderachter J.A. (2012). Claudin-1, Claudin-2 and Claudin-11 Genes Differentially Associate with Distinct Types of Anti-inflammatory Macrophages In vitro and with Parasite- and Tumour-elicited Macrophages In vivo. Scand. J. Immunol..

[103] Wang L., Liu X., Song X., Dong L., Liu D. (2019). MiR-202-5p promotes M2 polarization in allergic rhinitis by targeting MATN2. Int. Arch. Allergy Immunol..

[104] Wu D., Weng Y., Feng Y., Liang B., Wang H., Li L., Wang Z. (2021). Trem1 Induces Periodontal Inflammation via Regulating M1 Polarization. J. Dent. Res..

[105] Wu M.F., Lin C.A., Yuan T.H., Yeh H.Y., Su S.F., Guo C.L., Chang G.C., Li K.C., Ho C.C., Chen H.W. (2021). The M1/M2 spectrum and plasticity of malignant pleural effusion-macrophage in advanced lung cancer. Cancer Immunol. Immunother..

[106] Wu X., Giobbie-Hurder A., Liao X., Connelly C., Connolly E.M., Li J., Manos M.P., Lawrence D., McDermott D., Severgnini M. (2017). Angiopoietin-2 as a Biomarker and Target for Immune Checkpoint Therapy. Cancer Immunol. Res..

[107] Yan C., Zhou Q.-Y., Wu J., Xu N., Du Y., Li J., Liu J.-X., Koda S., Zhang B.-B., Yu Q. (2021). Csi-let-7a-5p delivered by extracellular vesicles from a liver fluke activates M1-like macrophages and exacerbates biliary injuries. Proc. Natl. Acad. Sci. USA.

[108] Yan J., Wu X., Yu J., Zhu Y., Cang S. (2020). Prognostic Role of Tumor Mutation Burden Combined With Immune Infiltrates in Skin Cutaneous Melanoma Based on Multi-Omics Analysis. Front. Oncol..

[109] Zhang A.Z., Yuan X., Liang W.H., Zhang H.J., Li Y., Xie Y.F., Li J.F., Jiang C.H., Li F.P., Shen X.H. (2021). Immune Infiltration in Gastric Cancer Microenvironment and Its Clinical Significance. Front. Cell Dev. Biol..

