# Supplementary material for: Transcriptomic Establishment of Pig Macrophage Polarization Signatures
Source: Curr Issues Mol Biol. 2023 Mar 12;45(3):2338–50. doi: 10.3390/cimb45030151 (PMC10047103; doi:10.3390/cimb45030151)
Supplement: Supplementary file 1 [file cimb-45-00151-s001.zip › Additional file/List of additional file.pdf]

List of additional files

Additional file1. Transcriptomic comparison of porcine macrophage phenotypes, M1 versus M2. List of all DEGs, enrichment pathways, and hubs identified by transcriptome analysis of M1 versus M2.

Additional file2. Transcriptomic comparison of porcine macrophage phenotypes, M1_IFN γ +LPS versus M1_GM-CSF. List of all DEGs, enrichment pathways, and hubs identified by transcriptome analysis of M1_IFNγ+LPS versus M1_GM-CSF.

Additional file3. Transcriptomic comparison of porcine macrophage phenotypes, M2_IL4+IL10 versus M2_M-CSF. List of all DEGs, enrichment pathways, and hubs identified by transcriptome analysis of M2_IL4+IL10 versus M2_M-CSF.

Additional file4. List of enrichment pathways identified by GSEA analysis of macrophage cultured *in vitro* vs pathogen-infected using GSEA software.
