# Supplementary material for: Transcriptomic Establishment of Pig Macrophage Polarization Signatures
Source: Curr Issues Mol Biol. 2023 Mar 12;45(3):2338–50. doi: 10.3390/cimb45030151 (PMC10047103; doi:10.3390/cimb45030151)
Supplement: Supplementary file 1 [file cimb-45-00151-s001.zip › Supplementary.pdf]

SUPPLEMENTARY MATERIAL- Figs and Tables

Supplementary Figs

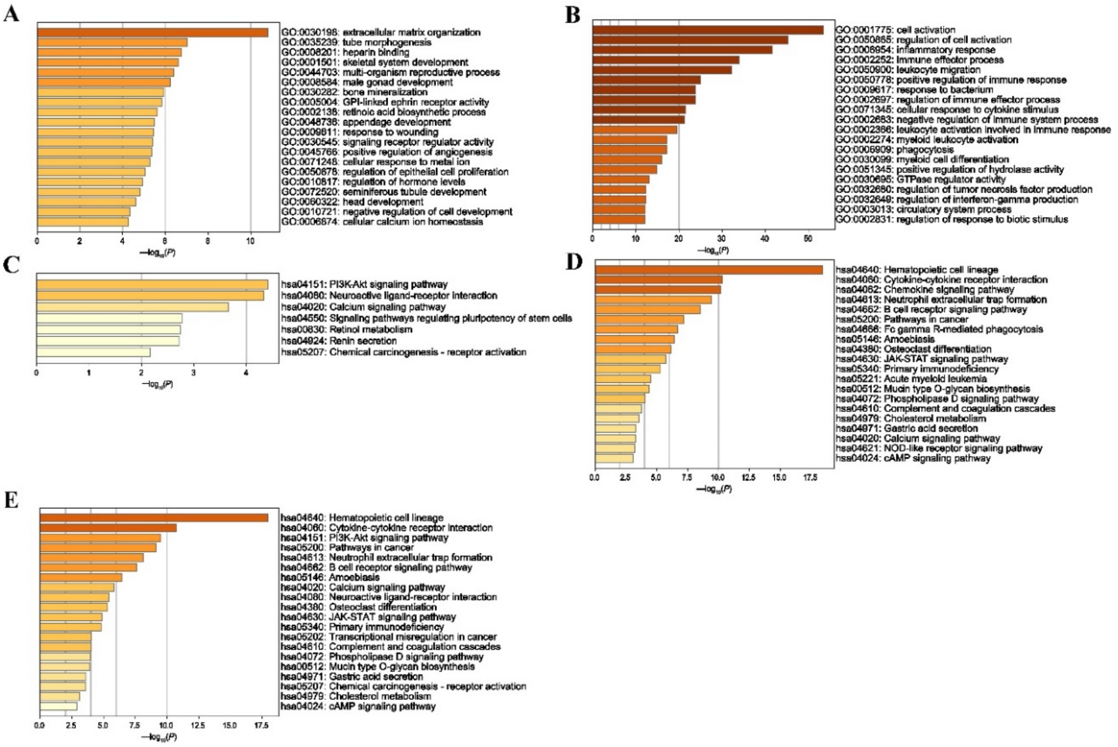

Supplementary Fig.1 The KEGG or GO Analysis of M1 and M2. (A) Top enriched GO biological processes of up-regulated genes. **(B)** Top enriched GO biological processes of down-regulated genes. **(C)** Top enriched KEGG biological processes of up-regulated genes. **(D)** Top enriched KEGG biological processes of down-regulated genes. **(E)** Top enriched KEGG biological processes of 730 genes.

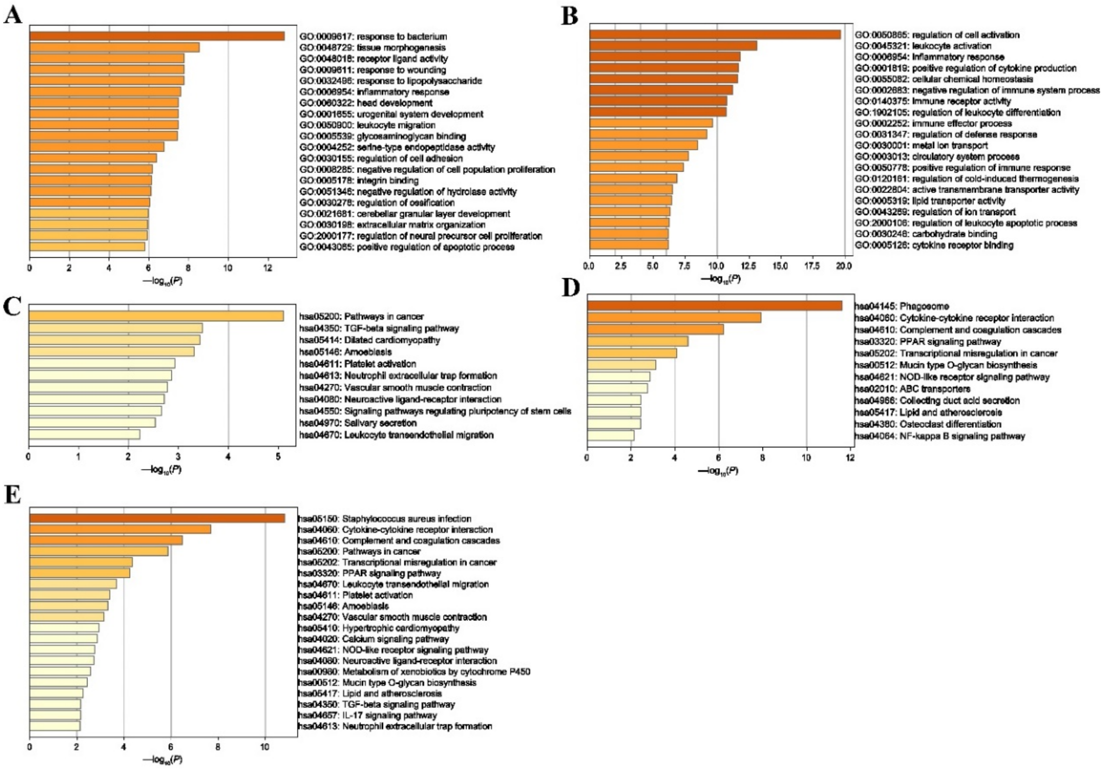

Supplementary Fig.2 The KEGG or GO Analysis of M1_IFN γ +LPS and M1_GM-CSF. (A) Top enriched GO biological processes of up-regulated genes. **(B)** Top enriched GO biological processes of down-regulated genes. **(C)**

Top enriched KEGG biological processes of up-regulated genes. **(D)** Top enriched KEGG biological processes of down-regulated genes. **(E)** Top enriched KEGG biological processes of 598 genes.

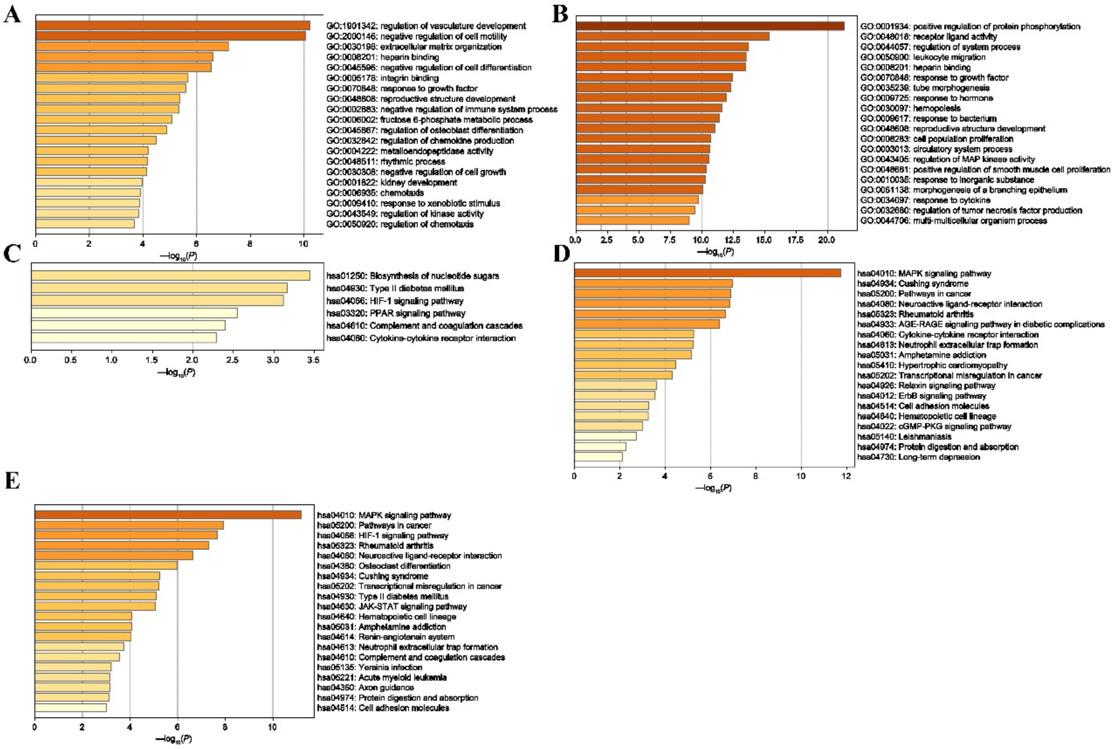

Supplementary Fig.3 The KEGG or GO Analysis of M2_IL4+IL10 and M2_M-CSF. (A) Top enriched GO biological processes of up-regulated genes. **(B)** Top enriched GO biological processes of down-regulated genes; **(C)** Top enriched KEGG biological processes of up-regulated genes. **(D)** Top enriched KEGG biological processes of down-regulated genes. **(E)** Top enriched KEGG biological processes of 332 genes.

Supplementary tables

S1 Table. Data output quality list

	Raw Reads	Clean Reads	Raw Base(G)	Clean Base(G)	Effective Rate (%)	Error Rate (%)	Q20(%)	Q30(%)	GC Content (%)
M1_IFN γ +LPS.3	55,816,079	54,627,974	16.74	16.39	97.87	0.04	97.3	91.98	53.09
M2_M-CSF.2	49,465,538	48,351,626	14.84	14.51	97.75	0.04	97.01	91.29	53.91
M1_IFN γ +LPS.1	75,618,946	73,922,737	22.69	22.18	97.76	0.04	97.23	92.13	54.19
M2_M-CSF.1	51,587,689	50,401,680	15.48	15.12	97.7	0.04	97.18	91.7	54.02
M2_M-CSF.4	49,014,356	48,379,317	14.7	14.51	98.7	0.04	96.56	90.06	53.42
M1_IFN γ +LPS.2	55,594,140	54,880,707	16.68	16.46	98.72	0.04	97.17	91.65	53.43
M1_GM-CSF.3	41,956,829	41,034,198	12.59	12.31	97.8	0.04	96.96	91.52	53.39
M1_IFN γ +LPS.4	56,583,524	55,406,603	16.98	16.62	97.92	0.04	97.38	92.24	53.66
M2_IL4+IL10.2	74,606,423	73,282,646	22.38	21.98	98.23	0.04	97.08	91.41	52.97
M1_GM-CSF.4	67,209,133	65,873,791	20.16	19.76	98.01	0.04	97.41	92.53	52.85
M1_GM-CSF.1	53,987,356	53,243,421	16.2	15.97	98.62	0.04	96.88	91.27	53.53
M2_IL4+IL10.1	61,546,698	60,779,788	18.46	18.23	98.75	0.04	96.86	90.85	54.12
M2_IL4+IL10.4	67,730,073	66,381,289	20.32	19.91	98.01	0.04	96.53	89.94	54.39
M1_GM-CSF.2	43,257,600	42,648,637	12.98	12.79	98.59	0.04	97.22	92.06	52.02

S2 Table. The observed marker genes of M1 and M2 macrophages

Gene	Cell Marker	References	Gene	Cell Marker	References
PADI4	M1 macrophage	(Cheng et al., 2021a)	HLA-DRA	M1 macrophage	(Camiolo et al., 2020; Wu et al., 2021b)
FCRL3	M1 macrophage	(Quan et al., 2021)	DLL4	M1 macrophage	(Pagie et al., 2018)
JAK3	M1 macrophage	(Quero et al., 2020)	IRF5	M1 macrophage	(Lv et al., 2021; Huang et al., 2022)
CXCR4	M1 macrophage	(Fang et al., 2021)	PTPRO	M1 macrophage	(Dong et al., 2021)
IL10RA	M1 macrophage	(Acosta et al., 2019)	MFAP4	M2 macrophage	(Huang et al., 2022)
TLR8	M1 macrophage	(Wu et al., 2021b)	PTH1R	M2 macrophage	(Zhang et al., 2021)
MARCO	M1 macrophage	(Talamonti et al., 2017; Sato et al., 2018)	CLDN11	M2 macrophage	(van den Bossche et al., 2012)
TLR1	M1 macrophage	(Müller et al., 2017)	AGTR1	M2 macrophage	(Cui et al., 2021)
TREM1	M1 macrophage	(Wu et al., 2021a)	COL9A3	M2 macrophage	(Liu et al., 2021)
CLEC7A	M1 macrophage	(Yan et al., 2021)	EEF1A2	M2 macrophage	(Lv et al., 2021; Tan et al., 2021)
TLR2	M1 macrophage	(Müller et al., 2017)	CRABP2	M2 macrophage	(Yan et al., 2020)
CLEC4E	M1 macrophage	(Inoue, 2017)	FGFR3	M2 macrophage	(Huang et al., 2022)
TRPM2	M1 macrophage	(Beceiro et al., 2017)	PTX3	M2 macrophage	(Huang et al., 2022)
S100A8	M1 macrophage	(LA et al., 2017)	CCL11	M2 macrophage	(Chen et al., 2019)
SLAMF1	M1 macrophage	(Al-Rubaie et al., 2018)	ALDH1A1	M2 macrophage	(Oliveira et al., 2010)
S100A9	M1 macrophage	(LA et al., 2017)	ENHO	M2 macrophage	(Sato et al., 2018)
CASP1	M1 macrophage	(Awad et al., 2017)	MATN2	M2 macrophage	(Wang et al., 2019a)
IRF8	M1 macrophage	(Guo et al., 2016)	ANGPT2	M2 macrophage	(Wu et al., 2017)
CD68	M1 macrophage	(Shapouri-Moghaddam et al., 2018)	HMOX1	M2 macrophage	(Seneviratne et al., 2021)
IL27	M1 macrophage	(Al-Rubaie et al., 2018; Chiba et al., 2018)	IL1RL1	M2 macrophage	(Wang et al., 2019b)
MYO1F	M1 macrophage	(Song et al., 2021)	CYP1A1	M2 macrophage	(Cui et al., 2020)
IL7R	M1 macrophage	(Joerink et al., 2011)	CD5L	M2 macrophage	(Sanjurjo et al., 2018)
INPP5D	M1 macrophage	(Jablonski et al., 2016)	POSTN	M2 macrophage	(Binnemars-Postma et al., 2018)
CCR7	M1 macrophage	(Joerink et al., 2011; Cheng et al., 2021b)	CCN3	M2 macrophage	(Chen et al., 2014)
VENTX	M1 macrophage	(Le et al., 2018)			

References

Acosta, J. R., Tavira, B., Douagi, I., Kulyté, A., Arner, P., Rydén, M., et al. (2019). Human-Specific Function of IL-10 in Adipose Tissue Linked to Insulin Resistance. *J. Clin. Endocrinol. Metab.* 104, 4552–4562. doi: 10.1210/jc.2019-00341.

Al-Rubaie, A., Wise, A. F., Sozo, F., De Matteo, R., Samuel, C. S., Harding, R., et al. (2018). The therapeutic effect of mesenchymal stem cells on pulmonary myeloid cells following neonatal hyperoxic lung injury in mice. *Respir. Res.* 19, 1–11.

Awad, F., Assrawi, E., Jumeau, C., Georgin-Lavialle, S., Cobret, L., Duquesnoy, P., et al. (2017). Impact of human monocyte and macrophage polarization on NLR expression and NLRP3 inflammasome activation. *PLoS One* 12, e0175336.

Beceiro, S., Radin, J. N., Chatuvedi, R., Piazuelo, M. B., Horvarth, D. J., Cortado, H., et al. (2017). TRPM2 ion channels regulate macrophage polarization and gastric inflammation during Helicobacter pylori infection. *Mucosal Immunol.* 10, 493–507. doi: 10.1038/mi.2016.60.

Binnemars-Postma, K., Bansal, R., Storm, G., and Prakash, J. (2018). Targeting the Stat6 pathway in tumor-associated macrophages reduces tumor growth and metastatic niche formation in breast cancer. *FASEB J.* 32, 969–978. doi: 10.1096/fj.201700629R.

Camiolo, G., Barbato, A., Giallongo, C., Vicario, N., Romano, A., Parrinello, N. L., et al. (2020). Iron regulates myeloma cell/macrophage interaction and drives resistance to bortezomib. *Redox Biol.* 36, 101611.

Chen, C., Perry, T. L., Chitko-McKown, C. G., Smith, A. D., Cheung, L., Beshah, E., et al. (2019). The regulatory actions of retinoic acid on M2 polarization of porcine macrophages. *Dev. Comp. Immunol.* 98, 20–33.

Chen, P. C., Cheng, H. C., Wang, J., Wang, S. W., Tai, H. C., Lin, C. W., et al. (2014). Prostate cancer-derived CCN3 induces M2 macrophage infiltration and contributes to angiogenesis in prostate cancer microenvironment. *Oncotarget* 5, 1595–1608. doi: 10.18632/oncotarget.1570.

Cheng, Y., Si, Y., Wang, L., Ding, M., Yu, S., Lu, L., et al. (2021a). The regulation of macrophage polarization by hypoxia-PADI4 coordination in Rheumatoid arthritis. *Int. Immunopharmacol.* 99, 107988. doi: 10.1016/j.intimp.2021.107988.

Cheng, Y., Si, Y., Wang, L., Ding, M., Yu, S., Lu, L., et al. (2021b). The regulation of macrophage polarization by hypoxia-PADI4 coordination in Rheumatoid arthritis. *Int. Immunopharmacol.* 99, 107988.

Chiba, Y., Mizoguchi, I., Furusawa, J., Hasegawa, H., Ohashi, M., Xu, M., et al. (2018). Interleukin-27 exerts its antitumor effects by promoting differentiation of hematopoietic stem cells to M1 macrophages. *Cancer Res.* 78, 182–194.

Cui, Y., Chen, F., Gao, J., Lei, M., Wang, D., Jin, X., et al. (2021). Comprehensive landscape of the renin-angiotensin system in Pan-cancer: a potential downstream mediated mechanism of SARS-CoV-2. *Int. J. Biol. Sci.* 17, 3795.

Cui, Z., Feng, Y., Li, D., Li, T., Gao, P., and Xu, T. (2020). Activation of aryl hydrocarbon receptor (AhR) in mesenchymal stem cells modulates macrophage polarization in asthma. *J. Immunotoxicol.* 17, 21–30.

Dong, H., Xie, C., Jiang, Y., Li, K., Lin, Y., Pang, X., et al. (2021). Tumor-Derived Exosomal Protein Tyrosine Phosphatase Receptor Type O Polarizes Macrophage to Suppress Breast Tumor Cell Invasion and Migration. *Front. cell Dev. Biol.*, 2731.

Fang, X. Y., Zhan, Y. X., Zhou, X. M., Wu, L. N., Lin, J., Yi, Y. T., et al. (2021). CXCL12/CXCR4 Mediates Orthodontic Root Resorption via Regulating the M1/M2 Ratio. *J. Dent. Res.*, 00220345211050324.

Guo, Y., Lin, C., Xu, P., Wu, S., Fu, X., Xia, W., et al. (2016). AGEs induced autophagy impairs cutaneous wound healing via stimulating macrophage polarization to M1 in diabetes. *Sci. Rep.* 6, 1–16.

Huang, Y., Li, Q., Hu, R., Li, R., and Yang, Y. (2022). Five immune-related genes as diagnostic markers for endometriosis and their correlation with immune infiltration. *Front. Endocrinol. (Lausanne).* 13, 1–15. doi: 10.3389/fendo.2022.1011742.

Inoue, T. (2017). M1 macrophage triggered by Mincle leads to a deterioration of acute kidney injury. *Kidney Int.* 91, 526–529. doi: 10.1016/j.kint.2016.11.026.

Jablonski, K. A., Gaudet, A. D., Amici, S. A., Popovich, P. G., and Guerau-de-Arellano, M. (2016). Control of the inflammatory macrophage transcriptional signature by miR-155. *PLoS One* 11, e0159724.

Joerink, M., Rindsjö, E., Van Riel, B., Alm, J., and Papadogiannakis, N. (2011). Placental macrophage (Hofbauer cell) polarization is independent of maternal allergen-sensitization and presence of chorioamnionitis. *Placenta* 32, 380–385.

LA, O., A, S., and JS, B. (2017). 乳鼠心肌提取 HHS Public Access. *Physiol. Behav.* 176, 139–148. doi: 10.1161/CIRCULATIONAHA.118.034165.Anti-angiogenic.

Le, Y., Gao, H., Bleday, R., and Zhu, Z. (2018). The homeobox protein VentX reverts immune suppression in the tumor microenvironment. *Nat. Commun.* 9, 1–10.

Liu, M., Li, F., Liu, B., Jian, Y., Zhang, D., Zhou, H., et al. (2021). Profiles of immune cell infiltration and immune-related genes in the tumor microenvironment of esophageal squamous cell carcinoma. *BMC Med. Genomics* 14, 1–11.

Lv, D., Wu, X., Chen, X., Yang, S., Chen, W., Wang, M., et al. (2021). A novel immune-related gene-based prognostic signature to predict biochemical recurrence in patients with prostate cancer after radical prostatectomy. *Cancer Immunol. Immunother.* 70, 3587–3602.

Müller, E., Christopoulos, P. F., Halder, S., Lunde, A., Beraki, K., Speth, M., et al. (2017). Toll-like receptor ligands and interferon-γ synergize for induction of antitumor M1 macrophages. *Front. Immunol.* 8, 1383.

Oliveira, L. J., McClellan, S., and Hansen, P. J. (2010). Differentiation of the endometrial macrophage during pregnancy in the cow. *PLoS One* 5, e13213.

Pagie, S., Gérard, N., and Charreau, B. (2018). Notch signaling triggered via the ligand DLL4 impedes M2 macrophage differentiation and promotes their apoptosis. *Cell Commun. Signal.* 16, 1–12.

Quan, Q., Xiong, X., Wu, S., and Yu, M. (2021). Identification of Immune-Related Key Genes in Ovarian Cancer Based on WGCNA. *Front. Genet.*, 2214.

Quero, L., Tiaden, A. N., Hanser, E., Roux, J., Laski, A., Hall, J., et al. (2020). miR-221-3p Drives the Shift of M2-Macrophages to a Pro-Inflammatory Function by Suppressing JAK3/STAT3 Activation. *Front. Immunol.* 10. doi: 10.3389/fimmu.2019.03087.

Sanjurjo, L., Aran, G., Téllez, É., Amézaga, N., Armengol, C., López, D., et al. (2018). CD5L promotes M2 macrophage polarization through autophagy-mediated upregulation of ID3. *Front. Immunol.* 9, 480.

Sato, K., Yamashita, T., Shirai, R., Shibata, K., Okano, T., Yamaguchi, M., et al. (2018). Adropin contributes to anti-atherosclerosis by suppressing monocyte-endothelial cell adhesion and smooth muscle cell proliferation. *Int. J. Mol. Sci.* 19, 1293.

Seneviratne, A., Cave, L., Hyde, G., Moestrup, S. K., Carling, D., Mason, J. C., et al. (2021). Metformin directly suppresses atherosclerosis in normoglycaemic mice via haematopoietic adenosine monophosphate-activated protein kinase. *Cardiovasc. Res.* 117, 1295–1308. doi: 10.1093/cvr/cvaa171.

Shapouri-Moghaddam, A., Mohammadian, S., Vazini, H., Taghadosi, M., Esmaeili, S. A., Mardani, F., et al. (2018). *Macrophage plasticity, polarization, and function in health and disease*. doi: 10.1002/jcp.26429.

Song, W.-M., Agrawal, P., Von Itter, R., Fontanals-Cirera, B., Wang, M., Zhou, X., et al. (2021). Network models of primary melanoma microenvironments identify key melanoma regulators underlying prognosis. *Nat. Commun.* 12, 1–14.

Talamonti, E., Pauter, A. M., Asadi, A., Fischer, A. W., Chiurchiù, V., and Jacobsson, A. (2017). Impairment of systemic DHA synthesis affects macrophage plasticity and polarization: implications for DHA supplementation during inflammation. *Cell. Mol. Life Sci.* 74, 2815–2826.

Tan, Y., Sun, R., Liu, L., Yang, D., Xiang, Q., Li, L., et al. (2021). Tumor suppressor DRD2 facilitates M1 macrophages and restricts NF-κB signaling to trigger pyroptosis in breast cancer. *Theranostics* 11, 5214.

van den Bossche, J., Laoui, D., Morias, Y., Movahedi, K., Raes, G., de Baetselier, P., et al. (2012). Claudin-1, Claudin-2 and Claudin-11 Genes Differentially Associate with Distinct Types of Anti-inflammatory Macrophages In vitro and with Parasite- and Tumour-elicited Macrophages In vivo. *Scand. J. Immunol.* 75, 588–598. doi: 10.1111/j.1365-3083.2012.02689.x.

Wang, L., Liu, X., Song, X., Dong, L., and Liu, D. (2019a). MiR-202-5p promotes M2 polarization in allergic rhinitis by targeting MATN2. *Int. Arch. Allergy Immunol.* 178, 119–127.

Wang, L., Liu, X., Song, X., Dong, L., and Liu, D. (2019b). MiR-202-5p Promotes M2 Polarization in Allergic Rhinitis by Targeting MATN2. *Int. Arch. Allergy Immunol.* 178, 119–127. doi: 10.1159/000493803.

Wu, D., Weng, Y., Feng, Y., Liang, B., Wang, H., Li, L., et al. (2021a). Trem1 Induces Periodontal Inflammation via Regulating M1 Polarization. *J. Dent. Res.*, 00220345211044681.

Wu, M.-F., Lin, C.-A., Yuan, T.-H., Yeh, H.-Y., Su, S.-F., Guo, C.-L., et al. (2021b). The M1/M2 spectrum and plasticity of malignant pleural effusion-macrophage in advanced lung cancer. *Cancer Immunol. Immunother.* 70, 1435–1450.

Wu, X., Giobbie-Hurder, A., Liao, X., Connelly, C., Connolly, E. M., Li, J., et al. (2017). Angiopoietin-2 as a Biomarker and Target for Immune Checkpoint Therapy. *Cancer Immunol. Res.* 5, 17–28. doi: 10.1158/2326-6066.CIR-16-0206.

Yan, C., Zhou, Q.-Y., Wu, J., Xu, N., Du, Y., Li, J., et al. (2021). Csi-let-7a-5p delivered by extracellular vesicles from a liver fluke activates M1-like macrophages and exacerbates biliary injuries. *Proc. Natl. Acad. Sci.* 118.

Yan, J., Wu, X., Yu, J., Zhu, Y., and Cang, S. (2020). Prognostic Role of Tumor Mutation Burden Combined With Immune Infiltrates in Skin Cutaneous Melanoma Based on Multi-Omics Analysis. *Front. Oncol.* 10, 2403.

Zhang, A. Z., Yuan, X., Liang, W. H., Zhang, H. J., Li, Y., Xie, Y. F., et al. (2021). Immune Infiltration in Gastric Cancer Microenvironment and Its Clinical Significance. *Front. Cell Dev. Biol.* 9.
